# Irradiation pasteurization of solid foods.

**DOI:** 10.3201/eid0402.980233

**Published:** 1998

**Authors:** S Moses, R C Brunham


3. Lin J, Smith MP, Chapin KC, Baik HS, Bennett GN, Foster JW. Mechanisms of acid resistance in enterohemorrhagic *Escherichia coli*. Appl Environ Microbiol 1996;62:3094-100.
4. Michino H, Araki K, Minami S, Takaya S, Sakai N. Investigation of large-scale outbreak of *Escherichia coli* O157:H7 infection among schoolchildren in Sakai City, 1996. In: Proceedings of the 32nd Joint Conference on Cholera and Related Diarrheal Diseases, U.S.-Japan Cooperative Medical Science Program(USJCMSP); 14-16 Nov 1996; Nagasaki University, Nagasaki, Japan. USJCMSP: 1996. p. 84-9.
5. Nathan R. American seeds suspected in Japanese food poisoning epidemic. Nat Med 1997;3:705-6.

### Irradiation Pasteurization of Solid Foods

**To the Editor:** Osterholm and Potter have made a strong case for irradiation pasteurization of solid foods that enter kitchens as raw agricultural commodities, such as meat, poultry, and seafood (1). Irradiation pasteurization was advocated to protect against foodborne diseases caused by common pathogens such as *Campylobacter*, *Cryptosporidium*, *Escherichia coli*, *Listeria*, *Salmonella*, and *Toxoplasma* (2). An additional rationale for irradiation pasteurization is bacterial resistance to antimicrobial drugs, a major health concern, which will undoubtedly increase in magnitude unless new approaches become available (3). The widespread use of antibiotics in animal husbandry may be the cause of some of this resistance, for example, in vancomycin-resistant enterococci associated with the agricultural use of glycopeptide antibiotics (4,5). Furthermore, resistance to glycopeptide antibitiotics can be transferred from enterococci to other gram-positive organisms, at least in the laboratory (6). Thus, resistant bacterial strains from animal sources may enter the human population through contaminated food without necessarily causing immediate disease but resulting in expanded human reservoirs of antimicrobial resistance through horizontal gene transfer (7). When such bacterial strains are subsequently transmitted to a susceptible person, serious disease could result, which would be exceedingly difficult to treat (8). Irradiation pasteurization of solid foods could reduce the magnitude of transfer of resistance genes through contaminated foods.

**Stephen Moses and Robert C. Brunham**
University of Manitoba, Winnipeg, Canada

### References

1. Osterholm MT, Potter ME. Irradiation pasteurization of solid foods: taking food safety to the next level. Emerg Infect Dis 1997;3:575-6.
2. Monk JD, Beuchat LR, Doyle MP. Irradiation inactivation of food-borne microorganisms. Journal of Food Protection 1995;58:197-208.
3. Gold HS, Moellering RC. Antimicrobial-drug resistance. N Engl J Med 1996;335:1445-53.
4. Bates J, Jordens JZ, Griffiths DT. Farm animals as a putative reservoir for vancomycin-resistant enterococcal infection in man. J Antimicrob Chemother 1994;34:507-14.
5. Van de Bogaard AE, Jensen LB, Stobberingh EE. Vancomycin-resistant enterococci in turkeys and farmers [letter]. N Engl J Med 1997;337:1558-9.
6. Leclerq R, Derlot E, Weber M, Duval J, Courvalin P. Transferable vancomycin and teicoplanin resistance in *Enterococcus faecium*. Antimicrob Agents Chemother 1989;33:10-5.
7. Davies J. Inactivation of antibiotics and the dissemination of resistance genes. Science 1994;264:375-82.
8. Swartz MN. Hospital-acquired infections: diseases with increasingly limited therapies. Proc Natl Acad Sci U S A 1994;91:2420-7.

### Emerging Infectious Diseases in Brazil

**To the Editor:** Hooman Momen's update on emerging infectious diseases in Brazil (1) appears to be based solely on notifiable disease data, which cannot adequately describe the current situation. Additional data in several areas may be useful.

Parasitic diseases: Dr. Momen's update restricts itself to protozoal diseases and does not distinguish between mucocutaneous and visceral leishmaniasis. Visceral leishmaniasis is in fact expanding in many suburban and urban areas in the northeast. Mucocutaneous leishmaniasis, after a small retreat following extensive deforestation, has made a comeback; and in many suburban areas in Rio de Janeiro and São Paulo, in the southeast, transmission is occurring, probably because of changes in sandfly ecology (1).

A helminthic disease of interest is mansoni schistosomiasis, which has been expanding its area of transmission, reaching over to Santa Catarina, in the south, to Pará in the north, expanding also westward, to Mato Grosso and Mato Grosso do Sul. The number of cases, as well as the associated illness, has possibly been reduced, but there is no doubt that the disease can be found in a much larger area than 20 years ago. Other emerging helminthiases of interest, albeit not of public health concern, are

